# Outbreak of NDM-5-Producing *Proteus mirabilis* During the COVID-19 Pandemic in an Argentine Hospital

**DOI:** 10.3390/antibiotics14060557

**Published:** 2025-05-29

**Authors:** Barbara Ghiglione, Ana Paula Rodriguez, María Sol Haim, Laura Esther Friedman, Nilton Lincopan, María Eugenia Ochiuzzi, José Alejandro Di Conza

**Affiliations:** 1 Instituto de Investigaciones en Bacteriología y Virología Molecular (IBaViM), Facultad de Farmacia y Bioquímica, Universidad de Buenos Aires, Buenos Aires 1113, Argentina; barbaraghiglione@gmail.com (B.G.); solhaim@gmail.com (M.S.H.); lauraestherfriedman@gmail.com (L.E.F.); 2 Consejo Nacional de Investigaciones Científicas y Técnicas (CONICET), Buenos Aires 1414, Argentina; 3 Hospital General de Agudos “Carlos G. Durand”, Buenos Aires 1405, Argentina; rodriguezapaula@gmail.com (A.P.R.); eugeoc@gmail.com (M.E.O.); 4 Unidad Operativa Centro Nacional de Genómica y Bioinformática, ANLIS Dr. Carlos G. Malbrán, Buenos Aires 1282, Argentina; 5 Department of Microbiology, Instituto de Ciencias Biomedicas, Universidade de Sao Paulo, Sao Paulo 05508-900, Brazil; lincopan@usp.br

**Keywords:** *Proteus mirabilis*, ST135, NDM-5, outbreak, RmtB, IncQ plasmid, biofilm formation

## Abstract

**Background:** During the COVID-19 pandemic, the emergence of multidrug-resistant (MDR) pathogens, driven by heightened antibiotic usage and device-associated infections, has posed significant challenges to healthcare. This study reports an outbreak of *Proteus mirabilis* producing NDM-5 and CTX-M-15 β-lactamases in a hospital in Buenos Aires, Argentina, from October 2020 to April 2021. To our knowledge, this represents the first documented outbreak of NDM-5-producing *P. mirabilis* in the country. **Methods:** A total of 82 isolates were recovered from 40 patients, with 41.5% from blood cultures and 18.3% from respiratory and urinary samples, among others. Antimicrobial susceptibility testing, PCR-based methods, and MALDI-TOF MS cluster analysis were conducted. Whole genome sequencing (WGS) was performed to characterize the MLST, resistome and plasmid content. Biofilm formation assays and in vitro rifampicin susceptibility tests were also conducted. **Result:** Most isolates exhibited resistance to carbapenems, cephalosporins, aminoglycosides, and fluoroquinolones, while retaining susceptibility to aztreonam. Genetic analysis confirmed the co-presence of the *bla*_NDM-5_ and *bla*_CTX-M-15_ genes. Clonal relationships was supported by PCR-based typing and MALDI-TOF MS cluster analysis. WGS revealed a resistome comprising 25 resistance genes, including *rmtB* and both β-lactamases, as well as the presence of an incomplete IncQ1 replicon associated with multiple resistance determinants. MLST classified this clone as belonging to ST135. Despite the biofilm-forming capacity observed across strains, rifampicin demonstrated potential for disrupting established biofilms at concentrations ≥32 µg/mL in vitro. The MDR profile of the outbreak strain significantly limited therapeutic options. **Conclusions:** This study highlights the growing threat of NDM-producing *P. mirabilis* in Argentina. The absence of surveillance cultures from the index case limits insights into the outbreak’s origin. These findings underscore the importance of integrating genomic surveillance into infection control protocols to mitigate the spread of MDR pathogens.

## 1. Introduction

*Proteus mirabilis*, a member of the *Enterobacterales* order, exhibits a widespread presence in both natural environments and the gastrointestinal tracts of humans and animals [1]. It is considered an opportunistic pathogen capable of causing nosocomial infections, particularly of the urinary tract, wounds, and respiratory tract, along with catheter-related infections and, less frequently, infections in the blood [2].

*P. mirabilis* naturally exhibits resistance to polymyxins, tetracyclines, tigecycline, and nitrofurantoin. Thereby, the acquisition of resistance genes against other antibiotic families such as β-lactams, aminoglycosides, and fluoroquinolones becomes a therapeutic challenge [3].

Beyond its role in acute infections, *P. mirabilis* exhibits a remarkable ability to form biofilms. Biofilm formation significantly enhances the bacterium’s resistance to antimicrobial agents and host immune responses, making infections caused by *P. mirabilis* challenging to treat. In the healthcare setting, biofilm-related infections are of particular concern, as they contribute to prolonged hospital stays, increased healthcare costs, and higher morbidity rates [4].

Moreover, *P. mirabilis* is able to produce urease, leading to increased urine alkalinity and the formation of crystalline biofilms. These biofilms often result in the encrustation of medical devices and the development of struvite and apatite stones, further complicating clinical management [5]. The presence of *P. mirabilis* biofilms on catheters poses significant clinical challenges. Catheter encrustation and blockage necessitate frequent replacements, increasing patient discomfort and healthcare costs. Furthermore, biofilm-associated bacteria can serve as a persistent source of infection, potentially leading to severe complications such as pyelonephritis and septicemia.

During the COVID-19 pandemic, the emergence of extremely resistant microorganisms was generally observed, and an increase in carbapenem resistance incidence was also documented, possibly linked to the heightened use of broad-spectrum antibiotics in the treatment of COVID-19 patients [6]. Concurrently, there has been an observed rise in the rate of device-associated infections in intensive care units, mainly in central vascular catheters and mechanical ventilation systems.

In recent years, there has been a significant increase in the prevalence of carbapenem-resistant *Enterobacterales*, with *P. mirabilis* isolates emerging as a particularly challenging concern within clinical environments [7]. This trend has significantly restricted the range of effective therapeutic strategies available. Resistance to carbapenems is mostly mediated through the production of carbapenemases such as KPC-2 and NDM-1 [8,9], although to a lesser extent, enzymes such as VIM or IMP contribute to this resistance phenotype [10].

Although different species of NDM-producing *Enterobacterales* were reported in Argentina, its documentation in *P. mirabilis* isolates was scarcely described. The NDM-5 variant was initially detected in an *E. coli* clinical isolate in 2018, and its prevalence has increased to date [11,12].

Local epidemiological data from the hospital indicated that, until August 2020, *P. mirabilis* isolates were predominantly susceptible to antibiotics, with only a small proportion producing extended-spectrum β-lactamases (ESBLs). Carbapenemase production had been confined to other *Enterobacterales* species and had not been detected in members of the *Proteae* tribe (Ochiuzzi, M.E., personal communication).

This study describes the first documented hospital outbreak in Argentina caused by a multidrug-resistant NDM-5-producing *Proteus mirabilis* clonal strain during the COVID-19 pandemic.

## 2. Results

### 2.1. Epidemiological and Clinical Characteristics of the P. mirabilis Outbreak

The outbreak lasted from October 2020 to April 2021 and involved 82 MDR *P. mirabilis* isolates from 40 hospitalized patients. The first strain was isolated on 29 September 2020, from a 61-year-old male patient with bacteremia. The demographic and clinical characteristics of the affected patients are summarized in Table 1. During the outbreak, *P. mirabilis* isolates were recovered from different clinical samples, including blood cultures (*n* = 34; 41.5%), tracheal aspirates/bronchoalveolar lavage fluid (*n* = 15; 18.3%), urine cultures (*n* = 15; 18.3%), catheters (*n* = 10; 12.2%), retrocultures (*n* = 5; 6.1%), and miscellaneous materials (*n* = 3; 3.6%). Surveillance isolates from anal swabs were not included. A total of 46.2% of the isolates were from patients hospitalized for COVID-19, while 26.8% were associated with other pathologies. The remaining patients were admitted for unknown reasons. The mean age was 52 years (range: 34–90 years), with 65% being male and 35% female.

### 2.2. Antimicrobial Susceptibility Testing (AST) and Carbapenemase Detection

The AST revealed that all 82 isolates were non-susceptible to imipenem (IMI, MIC ≥ 16 mg/L), meropenem (MEM, MIC ≥ 16 mg/L), cefotaxime (CTX, MIC ≥ 64 mg/L), ceftazidime (CAZ, MIC ≥ 64 mg/L), cefepime (FEP, MIC range 8 to ≥16 mg/L), piperacillin/tazobactam (PTZ, MIC range 16/4 to ≥124/4 mg/L), amikacin (AMK, MIC range 32 to ≥64 mg/L), gentamicin (GEN, MIC ≥ 16 mg/L), ciprofloxacin (CIP, MIC ≥ 4 mg/L), and trimethoprim/sulfamethoxazole (TMS, MIC ≥ 4/76 mg/L) but remained susceptible to aztreonam (AZT). In this study, “non-susceptible” refers to isolates categorized as either resistant or intermediate, according to CLSI 2020 breakpoints. Although the isolates were only susceptible to aztreonam, the XDR classification was not formally applied, as not all antimicrobial categories required for such designation were tested. Additionally, all *P. mirabilis* isolates displayed positive results for metallo-β-lactamase (MBL) production via synergy testing with EDTA. Molecular analysis via PCR confirmed the co-occurrence of *bla*_NDM_, encoding a carbapenemase, and *bla*_CTX-M-G1_, encoding an extended-spectrum beta-lactamase (ESBL). Subsequent sequencing of selected amplicons identified the presence of *bla*_NDM-5_ and *bla*_CTX-M-15_ variants.

### 2.3. Clonal Relationship

A subset of 28 isolates (one per patient) was examined to investigate the genetic relationship. These isolates were manually selected to represent the full duration of the outbreak and to include different types of clinical samples. Selection avoided repeated isolates from the same patient. Given that 40 patients were affected in total, this subset covers 70% of the cases, providing a representative sample for genetic relatedness analysis. Our results indicate minimal variation in amplification band profiles when performing REP- and ERIC-PCR, suggesting a close relationship among the strains (Figure S1). Consistent with the REP and ERIC results, most of these isolates (25/28) were grouped into a single MALDI-TOF cluster when a similarity cutoff of 85% was applied (Figure S2).

### 2.4. Biofilm Formation and Substrate-Specific Growth

**Figure 1:**
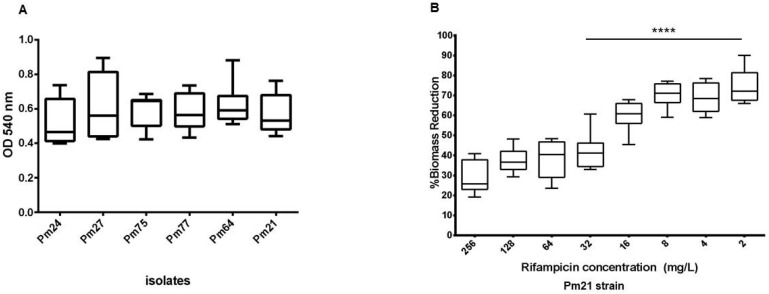
(**A**) Biofilm formation of *P. mirabilis* isolates obtained from different types of samples. (**B**) Effect of rifampicin on biofilm biomass of *P. mirabilis* Pm21 strain. Asterisks (****) indicate *p* < 0.0001 for biomass reduction measurement.

A subset of six *P. mirabilis* isolates was selected from the 28 non-duplicate isolates analyzed for clonal relatedness (Section 2.3) to evaluate biofilm formation. Quantitative assays demonstrated that all isolates formed moderate biofilms, as evidenced by biomass measurements. The ODs of the 48 h biofilms stained with CV ranged from 0.398 to 0.894 (Figure 1A, Table S1). The data analysis showed no significant statistical differences in biofilm formation among the isolates. Furthermore, biofilm formation ability was assessed on Foley urinary catheter materials, revealing that biofilms were readily established on latex catheters, whereas silicone catheters showed minimal biofilm development (Figure S3), highlighting the material-dependent nature of biofilm formation in *P. mirabilis*.

### 2.5. Impact of Rifampicin on Established Biofilms

The MIC against rifampicin of the six *P. mirabilis* isolates was 16 mg/L. The effect of rifampicin on preformed *P. mirabilis* biofilms was investigated using the microplates model against the Pm21 isolate. Following treatment with rifampicin at concentrations ranging from 256 to 2 mg/l, the minimum biofilm inhibitory concentration (MIC-b) was determined to be 128 mg/L. The minimum regrowth concentration (MRC), the lowest concentration preventing microbial recovery, was ≥256 mg/L. However, biomass quantification after rifampicin exposure revealed a significant reduction starting at 32 mg/L when compared to levels of the untreated controls, as confirmed by statistical analysis (Figure 1B, Table S2). These findings underscore the potential of rifampicin to disrupt *P. mirabilis* biofilms, albeit at relatively high concentrations.

### 2.6. Genome Analysis

Whole genome sequencing (WGS) analysis of isolate Pm21, recovered from a urine sample, revealed a genome size of 4,265,609 bp, with a GC content of 39.07%, 81 contigs (>1000 bp), and an N50 value of 140,806 bp. Pm21 displayed a multilocus sequence typing (MLST) profile consistent with sequence type (ST) 135 and a virulence profile corresponding to vST138. The resistome analysis predicted multiple acquired antimicrobial resistance genes, including *bla*_NDM-5_ and *bla*_CTX-M-15_ (β-lactam resistance), *rmtB*, *aadA1*, *aadA2*, *aadA5*, *aac(3)-IV*, *aac(6′)-Ib3*, *ant(3″)-Ia*, *aph(3′)-Ia*, *aph(6)-Id* (aminoglycoside resistance), *dfrA1*, *dfrA17*, *dfrA32* (trimethoprim resistance), *sul1* and *sul2* (sulfonamide resistance), *ere(A)* (macrolide resistance), *catA1* (chloramphenicol), and *tet(C)*, *tet(J)* (tetracycline resistance), among others (Table 2). Chromosomal point mutations were not detected since the PointFinder database is curated for species other than *Proteus* sp.

### 2.7. Genetic Analysis of Resistance Marker Environments

PlasmidFinder identified the IncQ1 replicon type as the only typable plasmid incompatibility group. The IncQ1 replicon was partially detected at the 5′ end of contig 49 (12,303 bp) with 100% identity, covering the first 524 bp of its total 796 bp length. The remaining portion of the replicon was not identified in any other contig within the Pm21 assembly or by mapping Illumina reads against its complete coding sequence. This suggests that contig 49 contains a truncated and likely non-functional IncQ1 replicon. BLAST (v1.4.0) analysis confirmed 100% identity and coverage of contig 49 with the chromosomes of *P. mirabilis* RGF134-1 (CP066833.1) and MPE0346 (CP053719.1), among others. In both animal-origin strains, this sequence is part of a chromosomal multidrug resistance genomic island (GI) containing *sul2*, *aac(3)-IId*, and *aph(6)-Id*, all located within contig 49 (Figure S4).

**Figure 2:**
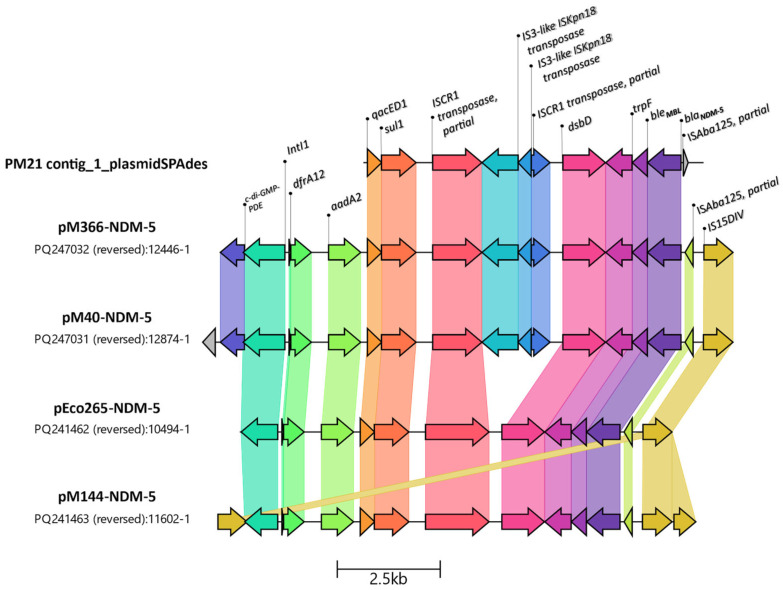
Comparison of the Pm21 contig and its genetic context with IncFII and multi-replicon IncFIB-HI1B plasmids, recently reported in Argentina, carrying *bla*_NDM-5_. The visualization was generated using Clinker (v 0.0.31). Arrows represent coding sequences, with homologous genes depicted in the same color and aligned based on sequence similarity.

A bleomycin resistance protein coded by *ble*_MBL_ was identified in contig 53 (6902 bp), downstream of the *bla*_NDM-5_ gene. A new assembly performed with plasmidSPAdes resulted in the generation of a longer contig (contig1_PlasmidSPAdes, 8258 bp, https://ri.conicet.gov.ar/handle/11336/252337, accessed on 25 May 2025). This allowed for the elucidation of the upstream region of *bla*_NDM-5_, where a truncated sequence of IS*Aba*125 was identified (Figure S5). The *bla*_NDM-5_ genetic context in Pm21 was as follows: (5′–3′: IS*Aba125*, partial sequence–*bla*_NDM-5_–*ble*_MBL_–*trpF*–*dsbD*–IS*91* family transposase–*qacEdelta*1–*sul*1). The IS*91* transposase coding gene was interrupted by IS*Kpn18*, exactly as observed in the IncFIB-IncHI1B plasmids, resulting in the novel genetic platform variant described by González-Espinosa et al. in plasmids pM366-NDM-5 and pM40-NDM-5 (Figure 2) [12].

BLAST analysis of contig 1_plasmidSPAdes revealed that the top three hits, with 100% coverage and identity, correspond to three *Klebsiella pneumoniae* recently deposited plasmids in Argentina: pM387-NDM5 (accession number CP168953.1), pM40-NDM5 (accession number PQ247031.1), and pM366-NDM5 (accession number PQ247032.1). The last two plasmids were fully characterized [12] (https://doi.org/10.1016/j.jgar.2024.10.258). All the remaining 97 hits listed after the BLAST analysis showed 100% identity but only 84% coverage, including *Escherichia coli* strain Ec265 plasmid pEco265-NDM5 (PQ241462.1) and *K. pneumoniae* plasmid pM144-NDM-5 (PQ241463), which have also been reported in the country (Figure 2).

The whole genome Pm21 assembly was compared to reference sequences using PLSDB with the mash screen search strategy to identify plasmids contained within the sample. The analysis yielded 32 entries, of which 12 contained the *bla*_NDM-5_ gene exclusively in *E. coli*. From these 12 plasmids, only those sequenced both by Illumina and Oxford Nanopore Technologies were further analyzed (10/12) (Table S3). Among the 10 remaining plasmids, 8 had a length of 10,494 bp. BLAST analysis of these plasmids showed 100% coverage and >99.98% similarity among them. Of the remaining two plasmids, one (LC744474.1, 10,687 bp) displayed 95% coverage and >99.98% similarity with the others, while the second plasmid (LC744490.1, 13,652 bp) showed 83% coverage and >99.95% nucleotide similarity (Figure S6). Of the 10 analyzed plasmids, only NZ_CP048374.1, with a length of 10,494 bp, had been previously published in a study assessing the occurrence of carbapenemase-producing *Enterobacteriaceae* (CPE) in freshwater samples from rivers, inland canals, and streams across Switzerland. This plasmid, named pC-F-163_C, was identified in extraintestinal pathogenic *E. coli* ST167, which was nontypeable by incompatibility group [13]. It showed 100% coverage and nucleotide similarity to *bla*_NDM-5-_carrying contig- from the Pm21 assembly generated by PlasmidSPAdes.

Additionally, the Pm21 strain also harbored the ESBL *bla*_CTX-M-15_ and the 16S ribosomal RNA methyltransferase *rmtB*, which were present in different contigs, 57 (4058 bp) and 67 (1961 bp), respectively. *bla*_CTX-M-15_ was found to have an upstream IS*1380*-like element belonging to the IS*Ecp1* family of transposases, while the *rmtB* gene was found in association with two downstream genes encoding a proton antiporter (*cdu2*) and the chaperonin GroEL, identical to other *rmtB*-containing *Enterobacterales* isolates from clinical samples (GenBank accession numbers CP050367, MN061455, MN007141) [11].

### 2.8. Phylogenetic Tree

To explore the diversity of ST135 and provide a broader geographic context to Pm21, a core genome SNP phylogenetic tree was generated using all genomic sequences of ST135 *P. mirabilis* available at PubMLST (October 2024). The full dataset containing 123 *P. mirabilis* ST135 isolates is available at Google Spreadsheet-Proteus mirabilis ST135 dataset. The resulting phylogenetic tree is available at Microreact (https://microreact.org/project/7rKjP28HQQkktYCjLbNbJu-proteuspm21st135, accessed on 25 May 2025). Most isolates are of clinical origin, but some were isolated from food and unknown sources. Besides Pm21, only one isolate carried *bla*_NDM-5_ (ABJNEB000000000.3), which was isolated from a human rectal swab. Our phylogenetic analysis revealed a high degree of dispersion among the *Proteus mirabilis* ST135 isolates. Pm21 did not cluster closely to any of the analyzed genomes. The closest related genome in this analysis was JZ9, which belongs to a Chinese isolate recovered from chicken meat. The prevalence of genes related to bacterial virulence factors, including urease (*ure*C), flagella genes (*flh*A, *fli*F, *fli*G, *fli*P, *fli*L, *flg*N), fimbriae (*mrp*A, *mrp*H, *uca*A, *pmf*A, *pmp*A, *pap*C, *pap*D, *pap*F, *pap*G, *pap*H), hemolysin (*hpm*AB), biofilm formation (*pst*C, *rcs*D), autotransporters (*pta*A, *aip*A), proteases (*zap*A), and siderophore-related (*nrp*R, *ire*A), was greater than 69% in all cases (*nrp*R showed the lowest prevalence 85/123). These results underscore the contribution of virulence determinants to the pathogenicity of ST135 *P. mirabilis*.

## 3. Discussion

This study documents an outbreak in an Argentine adult hospital attributed to *P. mirabilis* isolates co-producing NDM-5 MBL and CTX-M-15 ESBL. To date, reports of outbreaks caused by MDR *P. mirabilis* are scarce in the literature. Previously documented outbreaks have primarily involved *P. mirabilis* strains harboring carbapenemases such as NDM-1 [14] or VIM-4 [15], as well as ESBLs of the CTX-M-2, VEB-1, and TEM types [16,17,18].

While *bla*_NDM-5_ has been previously reported in *P. mirabilis* isolates from other countries, including China [19] and Austria [20], to our knowledge, this represents the initial documentation of *bla*_NDM-5_ in *P. mirabilis* in Argentina, and the first documented hospital outbreak caused by an NDM-producing *P. mirabilis* strain in the country.

Treatment of infections caused by such pathogens is often problematic due to their extensive drug resistance. The resistance pattern observed in these MDR *P. mirabilis* isolates generally reflects the hydrolysis spectrum of NDM-type enzymes, along with the presence of multiple aminoglycoside resistance markers and other antimicrobial resistance genes.

Despite carrying the CTX-M-15 ESBL, these isolates remained susceptible to aztreonam. Notably, this aztreonam susceptibility profile has also been observed in other clinical *P. mirabilis* isolates harboring ESBLs, AmpC, or carbapenemases, as previously reported by Shaaban et al. [21]. This antimicrobial agent, together with meropenem and piperacillin/tazobactam (when feasible), represented the main treatment options available for infections caused by this MDR *P. mirabilis* strain during the COVID-19 pandemic, a period marked by significant challenges in patient management. Following outbreak detection, the hospital’s infection control committee reinforced infection prevention measures, particularly hand hygiene practices and the change of personal protective equipment between patients. These strategies, already in place due to the COVID-19 pandemic, were intensified and played a key role in limiting further spread of the outbreak strain.

Since no surveillance cultures were performed on the index case at the time of hospital admission, it was not possible to determine whether this patient introduced the outbreak strain. Moreover, the fact that the first isolate was detected 50 days after hospitalization suggests that the strain was not acquired in the patient’s household or workplace but rather within the hospital from an unidentified source. In summary, the absence of surveillance cultures from the index case hampers the ability to trace the outbreak’s origins and fully characterize transmission dynamics. In addition, the absence of detailed clinical follow-up data, such as microbiological cure rates or patient outcomes, limited our ability to assess the full impact of the outbreak and the effectiveness of the treatments administered.

Given the high transmissibility and persistence of *P. mirabilis*-carrying NDM-5, effective infection control strategies are crucial for limiting further dissemination. Our findings suggest that, in line with repetitive sequence-based genomic amplification analyses, MALDI-TOF MS can aid in identifying clonally related groups of the opportunistic pathogen *P. mirabilis*. Supporting our results, previous studies have demonstrated the utility of MALDI-TOF MS as a first-line subtyping tool for the sensitive detection of potential dissemination events in hospital settings involving other opportunistic pathogens, such as *Serratia marcescens*, *Citrobacter freundii* [22], and *Enterobacter* species [23]. Although our clonal analysis relied primarily on REP-PCR, ERIC-PCR, and MALDI-TOF MS clustering, which are less discriminatory than whole genome sequencing-based approaches such as SNP analysis or cgMLST, the epidemiological data, including the unusually high frequency of carbapenem-resistant *P. mirabilis* isolates over a short time frame, support the occurrence of a clonal outbreak.

As observed in this study, *P. mirabilis* can cause catheter-associated urinary tract infections, largely due to its ability to form biofilms on catheter surfaces [24]. All six tested strains involved in this outbreak were classified as moderate biofilm producers in vitro. Notably, *P. mirabilis* is among the leading bacterial species responsible for biofilm-related infections associated with medical devices [25].

Interestingly, rifampicin exhibited potential for biofilm disruption at concentrations ≥32 µg/mL, although its clinical efficacy remains uncertain. Nwabor et al. reported that combining rifampicin with carbapenems enhanced its antibacterial activity and successfully eradicated established *Acinetobacter baumannii* biofilms [26]. Similarly, Amengol et al. demonstrated that colistin–rifampicin combinations effectively eradicated biofilms of both colistin-resistant and colistin-susceptible *Pseudomonas aeruginosa* [27]. These effects are likely related to the ability of rifampicin to penetrate the biofilm matrix (due to its lipophilic nature), inhibit RNA synthesis in metabolically active cells, and downregulate the expression of biofilm-associated genes [28]. However, to the best of our knowledge, no studies have specifically evaluated the effect of rifampicin on *P. mirabilis* biofilms.

We identified *bla*_NDM-5_ within a novel variant of the genetic platform recently described on multi-replicon IncFIB-IncHI1B plasmids in *K. pneumoniae* from Argentina. However, the antimicrobial resistance island containing *bla*_NDM-5_ in Pm21 could not be linked to any specific plasmid. BLAST analysis revealed that no more than 20% of pECO-265-NDM-5 and less than 15% of p366-NDM-5 were covered with Pm21 contigs (Figure S7A,B). pEco265-NDM-5 and pM144-NDM-5 belonged to the IncFII replicon type, encoding a MOB-F relaxase, whereas pM40-NDM-5 and pM366-NDM-5 were associated with multi-replicon IncFIB-IncHI1B plasmids, encoding a MOB-H relaxase [11,12]. Interestingly, neither the IncFII nor the IncHI1B replicons were detected in Pm21; however, the MOB-H relaxase was identified. *bla*_NDM-5_ has mainly been described in IncX3 plasmids as well as other Inc plasmids such as IncFII and IncI1, and in multi-replicon plasmids [29,30]. These findings indicate limited genetic similarity between circulating local plasmids and Pm21, raising the question of whether small, untypeable plasmids like pC-F-163 have already been circulating and integrating into local genetic platforms. This plasmid was determined to be highly similar at the nucleotide level (99–100%) to plasmids pM309-NDM5 and pM217_FII. Both plasmids were detected in nosocomial *E. coli* ST167 strains from a hematology ward in Myanmar between 2015 and 2016 [31]; (GenBank accession numbers AP018833.1 and AP018147.1, respectively). Additionally, pC-F-163_C showed 100% coverage and nucleotide similarity to plasmids circulating in Argentina, as described by González Espinosa et al. [12], including pM144-NDM-5, pEco265-NDM-5, pM40-NDM-5, and pM366-NDM-5, as well as to a *bla***_NDM-5_***-*carrying contig from a Pm21 assembly created by PlasmidSPAdes (Figure S8).

Pm21 also harbored the 16S ribosomal RNA methyltransferase *rmtB*, which was present in all plasmids described by González-Espinosa et al., except for pM144-NDM-5. Additionally, *bla*_CTX-M-15_ was detected in Pm21, as well as in the multi-replicon plasmids pM40-NDM-5 and pM366-NDM-5 [12].

The IncQ1 replicon was partially detected at the 5′ end of contig 49. We found that contig 49 displayed 100% identity and coverage with chromosomal multidrug resistance genomic islands (GI) in *P. mirabilis* of animal origin. According to the authors, they found six additional similar genomic islands, suggesting that the presence and spread of this IncQ1-harboring GI in *P. mirabilis* is not uncommon [32]. Interestingly, a BLAST search using the complete 796 bp *repA* sequence of IncQ1, filtered for *P. mirabilis*, yielded a total of 45 hits. Notably, all sequences with 66% coverage (~529 bp) were found in chromosomal DNA.

Our phylogenetic analysis demonstrated widespread dispersion among the *P. mirabilis* ST135 isolates, suggesting that MLST may not be a highly discriminatory tool for strain differentiation in this species. Given these findings, a more robust approach, such as core genome MLST (cgMLST), may be required for more precise strain typing, as proposed by Chen et al. [33]. However, despite its advantages, cgMLST has not yet been established as a standardized reference method for *P. mirabilis*, unlike in *Klebsiella* spp. This phylogenetic dispersion suggests the emergence of diverse NDM-producing *P. mirabilis* clones, reflecting a growing resistance threat. The global spread of multidrug-resistant strains, first reported in Italy and later in Poland, China, and Japan, underscores the need for effective prevention and control strategies [33].

## 4. Materials and Methods

### 4.1. Hospital Setting and Bacterial Isolates

This study was conducted at the Hospital General de Agudos “Carlos G. Durand” in Buenos Aires, Argentina, a 398-bed facility with intensive care units for adults and pediatric patients, intermediate care, a coronary unit, and general hospitalization wards. During the COVID-19 pandemic, the government implemented measures to optimize healthcare resources. Patient care was prioritized for SARS-CoV-2-infected individuals, as well as for emergency and critical cases. During periods of high bed occupancy, non-urgent medical interventions were postponed, based on medical assessment.

All bacterial isolates included in this study were recovered from clinical samples collected between October 2020 and April 2021 as part of routine diagnostic procedures. Samples were obtained by healthcare personnel in the corresponding inpatient services (e.g., ICU, medical clinic, surgery), following standard institutional protocols. Only clinical isolates were included in the analysis; no rectal swabs or surveillance cultures were used. For clonal analysis and further characterization, one isolate per patient was selected.

Following the initial detection of carbapenem-resistant *P. mirabilis*, a prospective survey was conducted to identify similar cases. Patient demographic data were saved from the laboratory database.

### 4.2. Bacterial Identification and Antimicrobial Susceptibility Testing (AST)

All clinical samples were processed at the hospital’s microbiology laboratory, following standard microbiological procedures. *Proteus mirabilis* identification was carried out using MALDI-TOF mass spectrometry (Vitek-MS^®^, bioMérieux, Marcy-l’Étoile, France), according to the manufacturer’s instructions. Antimicrobial susceptibility testing (AST) was performed using the Vitek 2C automated system (bioMérieux, Marcy-l’Étoile, France), also following the manufacturer’s protocols. MIC results were interpreted in accordance with the Clinical and Laboratory Standards Institute (CLSI) guidelines [34].

The minimum inhibitory concentration (MIC) of rifampicin was determined using the broth microdilution method in cation-adjusted Mueller–Hinton broth (MHB) [34], only for the six isolates included in the biofilm assays. Rifampicin was not assessed for therapeutic purposes, but rather to explore its potential activity against established biofilms.

### 4.3. Resistance Mechanisms

The production of carbapenemases was performed using the diffusion and synergy test, with meropenem (MEM), 10 µg, and imipenem (IPM), 10 µg discs (Laboratorio Britania, Buenos Aires, Argentina), interspersed with phenylboronic acid (PBA), 300 µg, and ethylenediaminetetraacetic acid (EDTA), 1 µmol discs, for assessing KPC and MBL, respectively [35].

Genotypic characterization of β-lactamase was carried out at Laboratorio de Resistencia Bacteriana (Facultad de Farmacia y Bioquímica, Universidad de Buenos Aires) using PCR amplification performed on the total DNA using specific primers for detection of metallo-carbapenemases (VIM, IMP and NDM) and extended spectrum β-lactamases (ESBLs) (CTX-M-G1, CTX-M-G2 and CTX-M-G9), under the conditions described previously [36,37]. Amplicons were sequenced on both strands using an ABI3730XL DNA Sequencer (Macrogen, Seoul, Republic of Korea).

### 4.4. Molecular Typing

The clonality of the isolates was determined by the homology relationships among fragments amplified by ERIC- (Enterobacterial Repetitive Intergenic Consensus) and REP- (Repetitive Extragenic Palindromic) PCR, according to the methods of Versalovic et al. [38].

Cluster analysis was performed using MALDI-TOF MS spectra with at least 110 peaks. A peak range from 3000 to 20,000 *m*/*z* was chosen for this clustering. Peaks were defined to be identical by applying a mass accuracy of 0.08% as the SARAMIS standard setting. Spectra were analyzed with a single link agglomerative clustering algorithm, applying the relative taxonomy analysis tool of SARAMIS premium software (v 4.1.1.0), to show the resulting dendrogram, with differences and similarities, in relative terms (percent matching masses). As a standard setting, the mass signal intensity was not considered in the cluster analysis [22].

### 4.5. Biofilm Formation Assays

A crystal violet (CV) assay was performed to evaluate the biofilm-forming ability of selected *P. mirabilis* isolates identified during the study period, using 96-well tissue-treated microplates [39]. Six isolates were selected as a representative subset of the outbreak strains, aiming to include the most common clinical sample sources: blood culture (three isolates, Pm24, Pm75, and Pm77), tracheal aspirate (Pm27), catheter (Pm64), and urine (Pm21, the sequenced isolate). Within these categories, the isolates were randomly chosen from different patients.

Briefly, isolates were subcultured on tryptic soy agar (TSA) for 24 h at 35 ± 2 °C, and a single colony from each was inoculated into lysogeny broth (LB) and grown overnight. The cultures were adjusted to a concentration of 10^6^ CFU/mL and resuspended in fresh LB. Aliquots (200 µL) of the standardized inoculum were dispensed into sterile, flat-bottom 96-well polystyrene microplates and incubated at 35 °C for 48 h. Following incubation, the wells were washed with phosphate-buffered saline (PBS) to remove non-adherent cells and stained with 0.01% (*w*/*v*) CV for 30 min. The stained biofilms were then washed with distilled water to remove excess dye. To quantify biofilm biomass, the bound CV was solubilized with 95% (*v*/*v*) ethanol, and absorbance was measured at 540 nm using a multimode plate reader. Each isolate was tested in octuplicate.

The biofilm-forming ability of the isolates was classified into four categories based on OD values, following the methods of Stepanovic et al. [40]:

Non-biofilm formers: OD ≤ ODcut;

Weak biofilm formers: ODcut < OD ≤ 2 × ODcut;

Moderate biofilm formers: 2 × ODcut < OD ≤ 4 × ODcut;

Strong biofilm formers: OD > 4 × ODcut.

ODcut was defined as the mean OD of the negative control plus its standard deviation.

To assess biofilm formation on a medical device, sections of latex and silicone Foley urinary catheter (2 cm in length) were cut under sterile conditions and incubated statically at 35 °C in 4 mL of LB containing 10^6^ CFU/mL of the Pm21 isolate. After 24 h, the catheter segments were removed, washed with PBS to eliminate non-adherent bacteria, and stained with CV, as described by Passerini de Rossi et al. (2012) [41]. The stained segments were then visually inspected for biofilm formation.

### 4.6. Biofilm Antimicrobial Susceptibility

Biofilm susceptibility testing of the Pm21 isolate was performed as described previously [39,42], with minor modifications. Briefly, aliquots (150 µL) of the standardized inoculum, as previously described, were added to the wells of a 96-well microplate and incubated at 35 °C for 48 h to allow for biofilm formation. After incubation, the medium was aseptically removed, the wells were washed with PBS, and 200 µL of rifampicin at two-fold serial dilutions (ranging from 256 mg/L to 2 mg/L) prepared in LB was added to the preformed biofilms.

Following overnight incubation, MIC-b was determined as the lowest antibiotic concentration that prevented the establishment of a planktonic bacterial population from the biofilm (i.e., no visible planktonic growth). The antibiotic solutions were then removed, the wells were washed, and 200 µL of LB was added. After 24 h of incubation at 35 °C, biofilm viability was assessed visually. The minimum regrowth concentration (MRC) was defined as the lowest antibiotic concentration at which bacteria failed to regrow [39,42]. Sterility and antibiotic-free controls were included in all experiments. Each condition was tested in octuplicate.

Additionally, the effect of rifampicin on biofilm biomass was assessed. Biofilms of Pm21 exposed to rifampicin (256 mg/L to 2 mg/L) were stained with CV, as previously described. Biomass reduction at each antibiotic concentration (eight wells per dilution) was quantified by calculating the ratio between the OD_540_ values of the treated and untreated biofilms.

### 4.7. Statistical Analysis

Differences in biofilm biomass between untreated and rifampicin-treated samples were assessed using the Kruskal–Wallis test, followed by Dunn’s multiple comparison test. Given the small sample size and uncertainty about the underlying distribution of the data, a non-parametric approach was selected as a conservative alternative to ANOVA. All *p*-values were calculated using one-tailed tests, with a significance level of 0.01. Statistical analyses and graphical representations were performed using GraphPad Software 6.01.

### 4.8. Whole Genome Sequencing Analysis

Whole genome sequencing (WGS) was performed on isolate Pm21, selected as a representative strain among the group of MDR *P. mirabilis* isolates recovered during the study period. This isolate was obtained from a urine sample. WGS of isolate Pm21 was performed through short reads on the Illumina NextSeq platform (Department of Microbiology of the Institute of Biomedical Sciences, Universidad de São Paulo, Brazil). Paired-end reads were de novo assembled into contigs using Unicycler (0.5.0+galaxy1) on the Galaxy platform (https://usegalaxy.eu/, accessed on 25 May 2025). Automated annotation was achieved with Prokka v.1.14.6, and the results were manually curated. The whole genome shotgun project of *P. mirabilis* Pm21 has been deposited in GenBank (SRA) under accession no. PRJNA1236692. The contig containing *bla*_NDM-5_ (contig 53) was subsequently annotated with BAKTA software (v 1.9.4) [43] to determine its genetic context. A new assembly was performed using plasmidSPAdes (v3.15.5) to obtain a longer contig. Sequence comparisons were performed using BLAST analysis. Strain typing was conducted using the Public Database for Molecular Typing and Microbial Genome Diversity (PubMLST; https://pubmlst.org/organisms/proteus-spp, accessed on 25 May 2025). The resistome was identified through ResFinder (22 March 2024) hosted on the Center for Genomic Epidemiology web page (http://genepi.food.dtu.dk/resfinder, accessed on 25 May 2025). Plasmid detection was carried out with PlasmidFinder v2.0.1, integrated into the staramr tool (v 0.11.0) [44]. Additionally, PLSDB, using the Mash screen search strategy, was employed to identify potential plasmid-related sequences and assess their similarity to those of previously reported plasmids [45,46,47]. To characterize genomic islands, in silico detection was performed using chromosomes deposited in NCBI and the IslandViewer 4 web server (http://www.pathogenomics.sfu.ca/islandviewer/, accessed on 25 May 2025) [48]. The graphical comparison of genomic islands was visualized with Clinker (https://cagecat.bioinformatics.nl/tools/clinker, accessed on 25 May 2025).

To explore ST135 diversity, a phylogenetic tree was built including Pm21 and all genomic sequences of ST135 *P. mirabilis* available at PubMLST (https://pubmlst.org/bigsdb?db=pubmlst_proteus_isolates&page=query&genomes=1, accessed on 25 May 2025). Briefly, core genome SNPs for ST135 genomes were determined using snippy (v4.6.0) [49], with the oldest available *P. mirabilis* genome (GB08, GCF_001617295.1) as a reference. Recombinant regions were removed using Gubbins (v3.3) [50]. Afterwards, an snp-only alignment was generated from the recombination-free Gubbins output using SNP-sites (v2.5.1) [51], which was used as the input for IQ-TREE (v1.6.12) [52]. The tree was built using the best fit model, determined with the function ‘-m TEST’ (TVM+F+ASC) and 1000 bootstraps. The tree was visualized with Microreact (https://microreact.org/, accessed on 25 May 2025) [53]. For all ST135 genomes, plasmid replicons, virulence, and antimicrobial resistance genes were detected using ABRicate, with different databases and thresholds: PlasmidFinder (default settings); a custom database with *P. mirabilis* virulence-related reference sequences; the Virulence Factor Database (VFDB) with an identity threshold of 70% and coverage of 60%; and ResFinder (default settings), respectively.

## 5. Conclusions

This study reports the first hospital outbreak in Argentina caused by *P. mirabilis* producing NDM-5, involving 40 patients during the COVID-19 pandemic. The outbreak strain exhibited an MDR profile, including *bla*_CTX-M-15_ and *rmtB* genes, and was confirmed as clonal by REP-/ERIC-PCR and MALDI-TOF MS analysis. All tested isolates formed moderate biofilms, and rifampicin reduced biofilm biomass in vitro at high concentrations. Genomic analysis revealed *bla*_NDM-5_ associated with the same genetic environment recently identified in *K. pneumoniae* in Argentina. No plasmid incompatibility groups other than partial IncQ1 were detected in this isolate. However, IncQ1 could not be linked to *bla*_NDM-5_.

These findings underscore the value of local epidemiological surveillance for early detection of unusual resistance profiles, which may indicate the beginning of an outbreak. Molecular characterization is essential to define such events and guide effective control measures. Although the transmission route was not determined, it may be related to the pandemic context, characterized by increased patient condition severity, antibiotic use, and invasive procedures. Strengthening genomic surveillance and infection control remains critical to limit the spread of high-risk MDR clones.

## Figures and Tables

**Table 1:** Demographic and clinical characteristics of 40 patients involved in the NDM-5-producing *Proteus mirabilis* outbreak.

Patient	Age	Sex	COVID Status	Sample Origin *	Date #	Sample Source (*n*)
1	61	M	positive	ICU	29 September 2020	blood culture (1), urine (1)
2	42	M	positive	MCU	1 October 2020	catheter (2), tracheal aspirate (2)
3	52	M	unknown	ICU	8 October 2020	catheter (1)
4	45	M	positive	ICU	20 October 2020	urine (1)
5	51	M	positive	ICU	23 October 2020	urine (1), tracheal aspirate (1)
6	37	F	negative	ICU	27 October 2020	catheter (1), blood culture (1)
7	60	M	positive	ICU	30 October 2020	blood culture (2)
8	46	M	positive	ICU	7 November 2020	catheter (1)
9	53	F	positive	ICU	8 November 2020	blood culture (2), tracheal aspirate (2)
10	57	M	positive	ICU	11 November 2020	blood culture (1), tracheal aspirate (1), urine (1)
11	45	M	negative	MCU	22 November 2020	urine (1), blood culture (1), catheter (1)
12	66	F	negative	GER	25 November 2020	blood culture (1)
13	47	F	unknown	MCU	25 November 2020	blood culture (1)
14	50	F	unknown	ICU	27 November 2020	blood culture (1)
15	53	F	negative	GS	2 December 2020	tracheal aspirate (1), miscellaneous (1)
16	51	M	negative	MCU	5 December 2020	blood culture (1)
17	55	F	unknown	MCU	6 December 2020	miscellaneous (1)
18	45	M	positive	MCU	20 December 2020	blood culture (1)
19	57	M	unknown	GER	7 January 2021	blood culture (1), urine (2)
20	65	F	negative	ICU	13 January 2021	tracheal aspirate (1)
21	60	M	negative	MCU	17 January 2021	urine (1)
22	43	M	negative	ICU	21 January 2021	urine (1)
23	48	F	positive	ICU	6 February 2021	blood culture (1), catheter (1)
24	90	M	negative	MCU	9 February 2021	miscellaneous (1)
25	45	M	unknown	ICU	10 February 2021	urine (1)
26	50	M	unknown	ICU	13 February 2021	blood culture (4), retroculture (1), catheter (1)
27	60	F	unknown	ICU	5 March 2021	blood culture (2)
28	71	M	unknown	MCU	10 March 2021	urine (1)
29	45	M	positive	ICU	12 March 2021	catheter (2)
30	44	F	negative	GS	24 March 2021	blood culture (3), retroculture (2)
31	54	M	positive	ICU	27 March 2021	tracheal aspirate (1), blood culture (2), urine (2)
32	57	M	positive	ICU	14 April 2021	tracheal aspirate (1)
33	64	M	negative	ICU	17 April 2021	blood culture (2), tracheal aspirate (1)
34	34	F	positive	ICU	18 April 2021	tracheal aspirate (1)
35	56	F	unknown	ICU	24 April 2021	blood culture (2), retroculture (2)
36	54	M	positive	MCU	26 April 2021	blood culture (2)
37	52	M	positive	ICU	27 April 2021	tracheal aspirate (1)
38	55	F	positive	ICU	27 April 2021	tracheal aspirate (1)
39	40	M	positive	ICU	29 April 2021	tracheal aspirate (1)
40	51	M	positive	ICU	30 April 2021	blood culture (2), urine (2)

* ICU: intensive care unit; MCU: medical clinic unit; GS: general surgery; GER: geriatrics. Samples labeled as “tracheal aspirate” may also include bronchoalveolar lavage fluid. Date # = date of the first isolate; (*n*) = number of isolates. The “miscellaneous” category includes isolates recovered from abdominal fluid, bone, and skin biopsy samples.

**Table 2:** Genomic characteristics of Pm21 isolate.

*Proteus mirabilis* Pm21 Assembly Metrics				
Characteristics		Details		
Genome size (bp)		4,265,609		
% GC content		39.07		
N50 (bp)		140,806		
**Resistome**				
**Gene**	**Predicted Phenotype**	**%Identity**	**%Coverage**	**HSP Length/Total Length**
*aac(3)-IId*	gentamicin	99.88	100	861/861
*aac(3)-IV*	gentamicin, tobramycin	100	100	777/777
*aadA1*	streptomycin	100	100	789/789
*aadA2*	streptomycin	99.88	97.92	802/819
*aadA5*	streptomycin	99.87	100	789/789
*aph(3* *″)-Ib*	streptomycin	100	100	804/804
*aph(3* *′)-Ia*	kanamycin	100	100	816/816
*aph(4)-Ia*	hygromycin	100	100	1026/1026
*aph(6)-Id*	kanamycin	100	100	837/837
*bla*_CTX-M-15_	ampicillin, ceftriaxone	100	100	876/876
*bla*_NDM-5_	ampicillin, amoxicillin/clavulanic acid, cefoxitin, ceftriaxone, meropenem	100	100	813/813
*bla*_OXA-1_	ampicillin	100	100	831/831
*bla*_OXA-2_	ampicillin	100	100	828/828
*bla*_TEM-1B_	ampicillin	100	100	861/861
*cat*	chloramphenicol	98.17	100.15	655/654
*catA1*	chloramphenicol	99.85	100	660/660
*catB3*	chloramphenicol	100	69.83	442/633
*dfrA1*	trimethoprim	100	100	474/474
*dfrA17-like* *	trimethoprim	100	86.92	412/474
*dfrA32-like* *	trimethoprim	100	86.92	412/474
*ere(A)*	erythromycin	99.84	100	1221/1221
*qacEdelta1*	resistance to antiseptics	100	84.68	282/333
*rmtB*	amikacin, gentamicin, kanamycin, streptomycin	100	100	756/756
*sul1*	sulfisoxazole	100	100	840/840
*sul2*	sulfisoxazole	100	100	816/816
*tet(C)*	tetracycline	99.66	100	1191/1191
*tet(J)*	tetracycline	99.08	100	1197/1197
**Plasmids**	IncQ1	100	65.83	524/796
**GenBank accession number**	Sequence Read Archive submission: PRJNA1236692.			
**Genome assembly**	https://ri.conicet.gov.ar/handle/11336/252337 (accessed on 25 May 2025)			

* Located on different contigs in the assembly.

## Data Availability

The whole genome shotgun project of *P. mirabilis* Pm21 has been deposited in GenBank under the Sequence Read Archive (SRA) accession no. submission: PRJNA1236692. The genome assembly (in Fasta format) used in this paper was deposited in the CONICET Digital Institutional Repository (https://ri.conicet.gov.ar/handle/11336/252337, accessed on 25 May 2025). Other data presented in this study are available upon request from the corresponding author.

## Supplementary Materials

The following supporting information can be downloaded at: https://www.mdpi.com/article/10.3390/antibiotics14060557/s1, Figure S1: Electrophoretic profiles of NDM-5-producing *P. mirabilis* isolates. Figure S2: Dendrogram of the MALDI-TOF spectra of *P. mirabilis* isolates using SARAMIS. Figure S3: Biofilm formation by *Proteus mirabilis* on different catheter materials. Figure S4: Linear comparison diagram of multi-drug resistance genomic island of RGF134-1, MPE0346 and contig 49 of Pm21. Figure S5. Comparison of *bla*_NDM-5_ genetic context in *Proteus mirabilis* Pm21. Figure S6. Nucleotide similarity and coverage of plasmids from PLSDB that displayed high identity with *P. mirabilis* Pm21 assembly and contained *bla*_NDM-5_. Figure S7. (A). Comparison among pECO-265-NDM-5 (used as a template), pM144-NDM-5, and the Pm21 assembly. BLAST analysis revealed no more than 20% coverage between Pm21 and pECO-265-NDM-5. Graphics were generated using Proksee, with relevant features annotated using CARD and mobileOG-DB. (B). Comparison among pM40-NDM-5 (used as a template), pM366-NDM-5, pECO-265-NDM-5 and the Pm21 assembly. BLAST analysis revealed less than 15% with pM40-NDM-5 and p366-NDM-5. Only CDSs identified by gene names using Bakta were included in the graphic to simplify comprehension. Figure S8. Nucleotide similarity and coverage of plasmid NZ_CP048374.1 (10,494 bp) with plasmids circulating in Argentina, including pM144-NDM-5, pEco265-NDM-5, pM40-NDM-5, and pM366-NDM-5, as well as with the Pm21 contig assembled using PlasmidSPAdes, all showing 100% coverage and nucleotide similarity. Table S1: Biofilm formation (OD_540_ values) of *Proteus mirabilis* isolates obtained from different patients and different types of samples (Pm24, Pm75* and Pm77* from blood cultures, Pm27 from tracheal aspirate, Pm64* from catheter and Pm21 from urine). Table S2: Effect of rifampicin on biofilm biomass (OD_540_ values) of *Proteus mirabilis* Pm21 strain. Table S3: Plasmids from PLSDB that displayed high identity with *Proteus mirabilis* Pm21 assembly and contained *bla*_NDM-5_.

## Abbreviations

The following abbreviations are used in this manuscript:

AMK	amikacin
AST	antimicrobial susceptibility testing
AZT	aztreonam
CAZ	ceftazidime
CPE	carbapenemase-producing Enterobacteriaceae
CFU	colony forming unit
CIP	ciprofloxacin
CLSI	Clinical and Laboratory Standards Institute
CTX	cefotaxime
CV	crystal violet
EDTA	ethylenediaminetetraacetic Acid
ERIC	Enterobacterial Repetitive Intergenic Consensus
ESBL	extended spectrum β-lactamase
FEP	cefepime
GEN	gentamicin
GI	genomic island
IPM	Imipenem
LB	lysogeny broth
MALDI-TOF MS	matrix-assisted laser desorption/ionization time-of-flight mass spectrometry
MB	minimum biofilm MIC (assumed: MIC-b)
MBL	metallo-β-lactamase
MDR	multidrug resistant
MEM	meropenem
MIC	minimum inhibitory concentration
MIC-b	minimum inhibitory concentration in the biofilm plate
MLST	multilocus sequence typing
MRC	minimum regrowth concentration
OD	optical density
PBA	phenylboronic acid
PBS	phosphate-buffered saline
PTZ	piperacillin/tazobactam
REP-PCR	repetitive extragenic palindromic PCR
ST	sequence type
TMS	trimethoprim/sulfamethoxazole
TSA	tryptic soy agar
TSB	tryptic soy broth
WGS	whole genome sequencing
cgMLST	core genome multilocus sequence typing
