# Supplementary material for: Outbreak of NDM-5-Producing Proteus mirabilis During the COVID-19 Pandemic in an Argentine Hospital
Source: Antibiotics (Basel). 2025 May 29;14(6):557. doi: 10.3390/antibiotics14060557 (PMC12189985; doi:10.3390/antibiotics14060557)

Supplementary material

Outbreak of NDM-5-producing *Proteus mirabilis* during the COVID-19 Pandemic in an Argentine Hospital

Barbara Ghiglione[1,2#], Ana Paula Rodriguez[3#], María Sol Haim[1,4], Laura Friedman[1], Nilton Lincopan[5], María E. Ochiuzzi[3], José Di Conza[1,2*]

Figure S1: Electrophoretic profiles of NDM-5-producing *P. mirabilis* isolates. (A) REP-PCR profiles; (B) ERIC-PCR profiles. Lanes are numbered with the odd-numbered IDs of 14 isolates selected from a total of 28 epidemiologically distinct, one-per-patient samples. Only odd-numbered isolate profiles are shown; the 14 even-numbered isolates exhibited indistinguishable banding patterns (data not shown). Isolate 21 was randomly selected for subsequent whole-genome sequencing and deeper genomic analysis.

A) REP-PCR

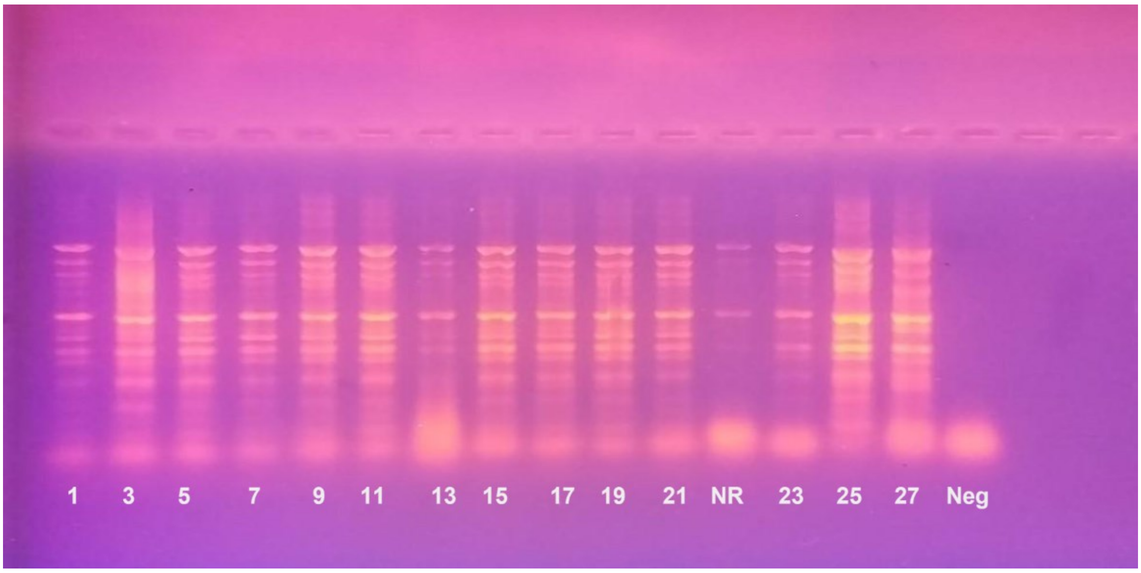

B) ERIC-PCR

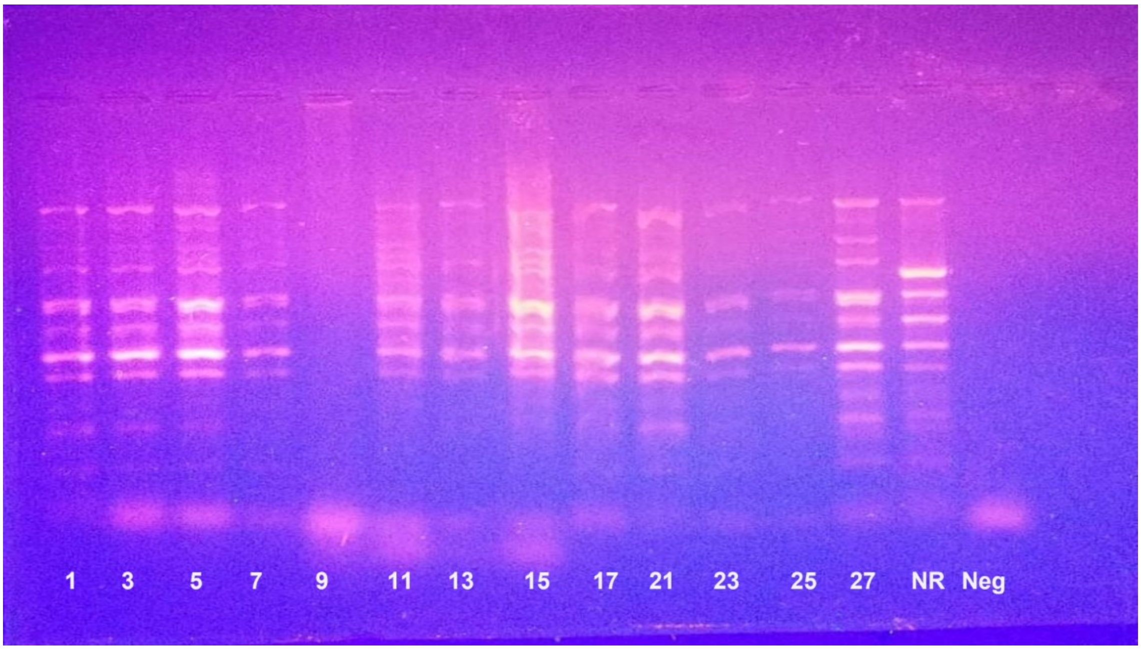

Figure S2: Dendrogram of the MALDI-TOF spectra of *P. mirabilis* isolates using SARAMIS. Isolate numbers (1–28) correspond to the same 28 epidemiologically distinct, one-per-patient samples previously analyzed by REP- and ERIC-PCR. The blue rectangle indicates clusters of outbreak isolates showing a mass spectrum similarity of ≥ 85%. Isolates 6, 17 and 19 displayed similarity of ≥ 80%.

Taxonomy relative

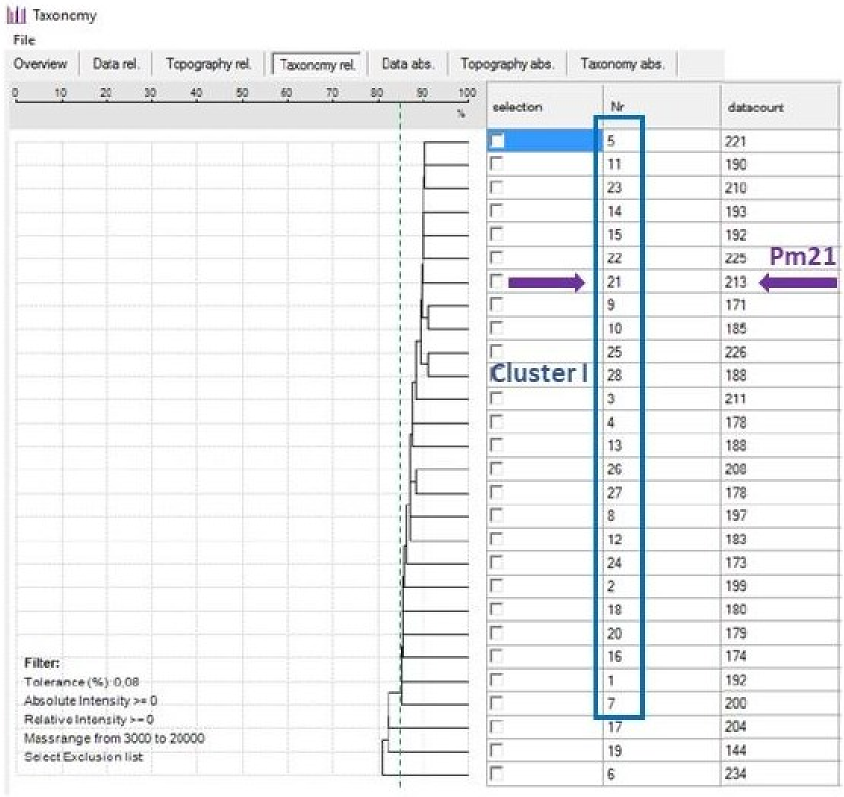

Table S1: Biofilm formation (OD_{540} values) of *Proteus mirabilis* isolates obtained from different patients and different types of samples (Pm24, Pm75* and Pm77* from blood cultures, Pm27 from tracheal aspirate, Pm64* from catheter and Pm21 from urine).

	**Pm 24**	**Pm27**	**Pm75***	**Pm77***	**Pm64***	**Pm21**
	0,7365	0,5827	0,6487	0,6968	0,8815	0,7632
	0,6776	0,6131	0,6862	0,7347	0,6794	0,6925
	0,5073	0,4323	0,6423	0,5460	0,5739	0,5286
	0,4241	0,5379	0,6475	0,4937	0,6086	0,4421
	0,4093	0,4248	0,4902	0,5064	0,5506	0,4733
	0,4214	0,4602	0,4229	0,4324	0,5393	0,5005
	0,3986	0,8942	0,5342	0,5828	0,5118	0,5333
	0,5945	0,8808	0,6522	0,6676	0,6571	0,6404
**Mean**	**0,5212**	**0,6033**	**0,5905**	**0,5826**	**0,6253**	**0,5717**
**Std. Deviation**	**0,1327**	**0,1882**	**0,09532**	**0,1076**	**0,1185**	**0,1139**

*Isolates marked with an asterisk were originally numbered according to the patient protocol used for the biofilm assay; however, they correspond to the following isolate IDs (used throughout the study, including in the REP-/ERIC-PCR profiles and the MALDI-TOF dendrogram): Pm64 → Isolate 4, Pm75 → Isolate 5, and Pm77 → Isolate 7.

Table S2: Effect of rifampicin on biofilm biomass (OD_{540} values) of *Proteus mirabilis* Pm21 strain.

Rifampicin concentration (mg/L)								Control
**256**	**128**	**64**	**32**	**16**	**8**	**4**	**2**	**0**
0,2201	0,2916	0,3282	0,4679	0,42450	0,4551	0,53000	0,5074	0,8773
0,3151	0,2489	0,2149	0,2577	0,34966	0,5894	0,60370	0,5144	0,7750
0,1870	0,2705	0,3369	0,3613	0,50810	0,5486	0,52490	0,6032	0,7779
0,1745	0,3244	0,3722	0,3084	0,50510	0,5462	0,54660	0,5359	0,6506
0,2082	0,3707	0,3679	0,3373	0,45320	0,5935	0,45397	0,6343	0,9128
0,3145	0,2714	0,2495	0,2541	0,47210	0,5670	0,51160	0,5358	0,6954
0,1886	0,3218	0,2954	0,2879	0,46560	0,5062	0,46600	0,6926	0,7622
0,1481	0,2253	0,1813	0,3266	0,52260	0,5280	0,60070	0,5742	0,7031

Figure S3: Biofilm formation by *Proteus mirabilis* on different catheter materials. Representative images of Foley urinary catheters after incubation with *P. mirabilis* isolate (Pm21), showing dense biofilm formation on latex catheters (left) and minimal biofilm on silicone catheters (right). These findings illustrate the material-dependent variability in biofilm development.

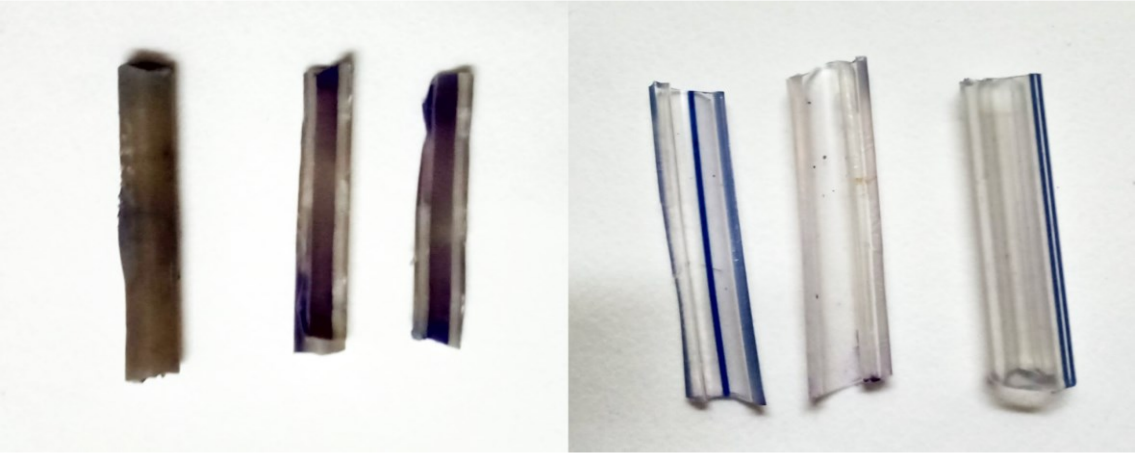

Figure S4: **Linear comparison diagram of multi-drug resistance genomic island of RGF134-1, MPE0346 and contig 49 of Pm21.** Following BLAST analysis, we confirmed that contig 49 maps with 100% identity to part of multidrug resistance (MDR) chromosomal genomic islands of *Proteus mirabilis* strains RGF134-1 and MPE0346.

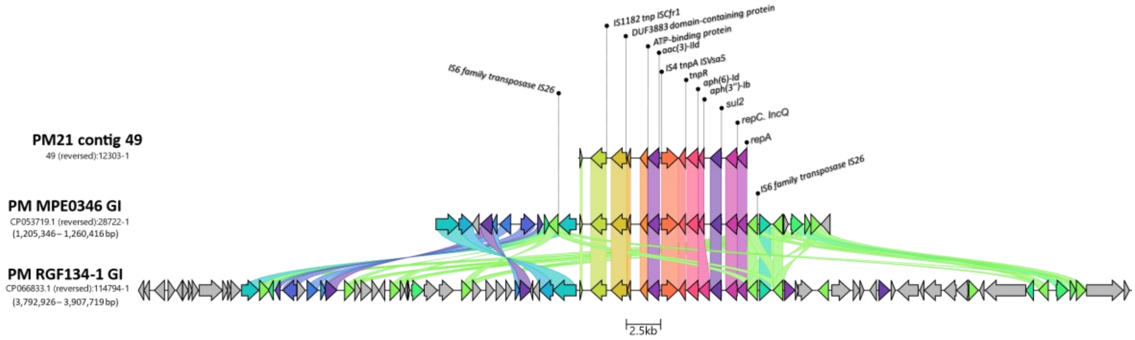

Figure S5. **Comparison of *bla*NDM-5 genetic context in *Proteus mirabilis* Pm21.** The genetic context of ***bla*NDM-5** was analyzed in two assemblies: contig 53 (6,902 bp, Unicycler) and contig 1 (8,258 bp, contig1_PlasmidSPAdes). Annotation revealed the *ble*MBL gene downstream of *bla*NDM-5 and a truncated sequence of ISAba125 upstream in the PlasmidSPAdes assembly. Graphics were generated using Clinker.

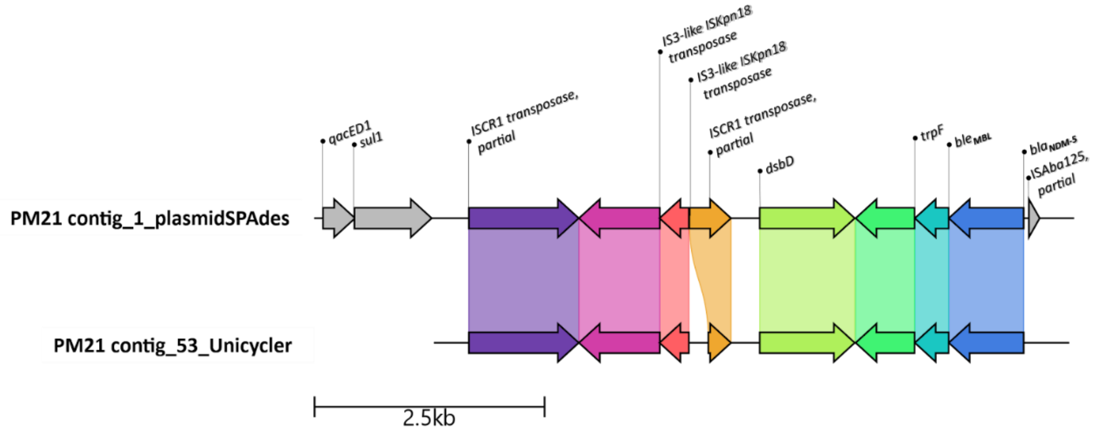

Table S3: Plasmids from PLSDB that displayed high identity with Proteus mirabilis Pm21 assembly and contained *bla*$_{NDM-5}$										
**Accession**	Topology	**blaNDM-5**	**plasmidfinder, MOB-Typer and pMLST**	Identity	**shared_hashes**	**Length**	sequencing technology	**Species**	ASSEMBLY_ACC	BIOSAMPLE_ACC
CP101345.1	linear	no	non-mobilizable	0.998746	974	2640		*Salmonella enterica*	GCA_024664475.1	SAMN29636373
CP101346.1	linear	no	non-mobilizable, IncQ	1	1000	1746		*Salmonella enterica*	GCA_024664475.1	SAMN29636373
CP101364.1	linear	no	non-mobilizable	0.99776	954	1268		*Salmonella enterica*	GCA_024664955.1	SAMN29636369
LC744442.1	circular	yes	non-mobilizable	0.993118	865	10494	MinION; Illumina MiSeq	*Escherichia coli*		
LC744459.1	circular	yes	non-mobilizable	0.993118	865	10494	MinION; Illumina MiSeq	*Escherichia coli*		
LC744474.1	circular	yes	non-mobilizable	0.993118	865	10687	MinION; Illumina MiSeq	*Escherichia coli*		
LC744476.1	circular	yes	non-mobilizable	0.993118	865	10494	MinION; Illumina MiSeq	*Escherichia coli*		
LC744490.1	circular	yes	non-mobilizable	0.993877	879	13652	MinION; Illumina MiSeq	*Escherichia coli*		
LC744501.1	circular	yes	non-mobilizable	0.993118	865	10494	MinION; Illumina MiSeq	*Escherichia coli*		
LC744505.1	circular	yes	non-mobilizable	0.993118	865	10494	MinION; Illumina MiSeq	*Escherichia coli*		
LC744510.1	circular	yes	non-mobilizable	0.993008	863	10494	MinION; Illumina MiSeq	*Escherichia coli*		
LC807778.1	circular	yes	non-mobilizable	0.993172	866	10494	MinION; Illumina NovaSeq 6000	*Escherichia coli*		
NZ_AJ437107.1	circular	no	non-mobilizable	0.998008	959	1091		*Escherichia coli*		SAMN14229489
NZ_CP048374.1	circular	yes	non-mobilizable	0.993227	867	10494	Oxford Nanopore/illumina minniseq	*Escherichia coli*	GCF_010365505.1	SAMN13951917
NZ_CP079667.1	circular	no	non-mobilizable	0.997159	942	2186		*Klebsiella pneumoniae*	GCF_019317185.1	SAMN20130620
NZ_CP079814.1	circular	no	non-mobilizable	0.996601	931	2710		*Klebsiella pneumoniae*	GCF_019334705.1	SAMN20115065
NZ_CP079817.1	circular	no	non-mobilizable	1	1000	1657		*Klebsiella pneumoniae*	GCF_019334705.1	SAMN20115065
NZ_CP084054.1	circular	no	non-mobilizable	0.996703	933	1355		*Klebsiella pneumoniae*	GCF_020215625.1	SAMN20114092
NZ_CP084904.1	circular	yes	non-mobilizable	0.998058	960	4363	Oxford Nanopore PromethION	*Escherichia coli*	GCF_020535205.1	SAMN21906625
NZ_CP086290.1	circular	no	non-mobilizable	0.999184	983	6874		*Citrobacter freundii*	GCF_020809005.1	SAMN21601121
NZ_CP091568.1	circular	no	non-mobilizable, IncQ	0.998795	975	7942		*Salmonella enterica*	GCF_023330865.1	SAMN16419718
NZ_CP127504.1	circular	no	non-mobilizable	0.996703	933	3431		*Klebsiella pneumoniae*	GCF_036452035.1	SAMN35640681
NZ_CP138830.1	circular	no	non-mobilizable	0.99771	953	2440		*Klebsiella pneumoniae*	GCF_033868275.1	SAMN38040199
NZ_CP148046.1	circular	no	non-mobilizable	0.994995	900	1860		*Morganella morganii*	GCF_037482145.1	SAMN40447594
NZ_CP149158.1	circular	no	non-mobilizable	0.992235	849	1924		*Salmonella enterica*	GCF_037824785.1	SAMN40440243
NZ_CP149182.1	circular	no	non-mobilizable, IncQ	1	1000	4386		*Salmonella enterica*		
NZ_CP149183.1	circular	no	non-mobilizable	0.999329	986	1916		*Salmonella enterica*		
NZ_KF220658.1	circular	no	non-mobilizable	0.999087	981	1634		*Klebsiella pneumoniae*		SAMN14225988
NZ_MN370929.1	circular	no	non-mobilizable	0.99731	945	3579		*Klebsiella pneumoniae*		
NZ_OR597496.1	circular	yes	non-mobilizable	1	1000	3113	illumina	*Escherichia coli*		
NZ_OZ040720.1	circular	no	non-mobilizable	0.995258	905	2630		*Escherichia coli*		SAMEA9068036
NZ_OZ040767.1	circular	no	non-mobilizable, IncQ	0.998697	973	7942		*Escherichia coli*		SAMEA9068048

Figure S6. Nucleotide similarity and coverage of plasmids from PLSDB that displayed high identity with *P. mirabilis* Pm21 assembly and contained *bla*$_{NDM-5}$. (A) Heatmap showing pairwise nucleotide similarity among the 10 small plasmids carrying *bla*$_{NDM-5}$. Similarity values were obtained using BLAST, with darker colors indicating higher similarity percentages. Plasmids with a length of 10,494 bp exhibit nearly identical sequences (>99.98% similarity), while LC744474.1 (10,687 bp) and LC744490.1 (13,652 bp) display slightly lower similarity values. (B) Heatmap illustrating coverage percentages of the same plasmids. Coverage was calculated based on BLAST alignment results, where darker shades represent higher coverage.

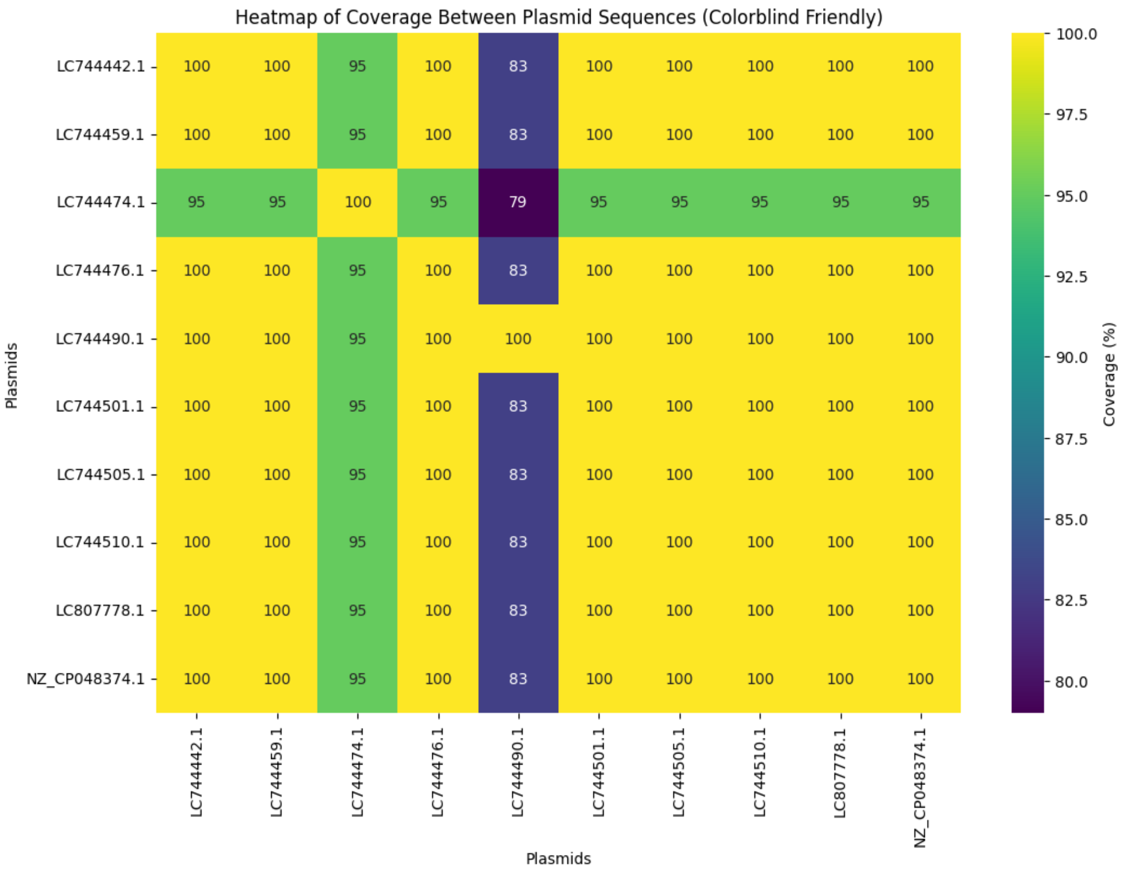

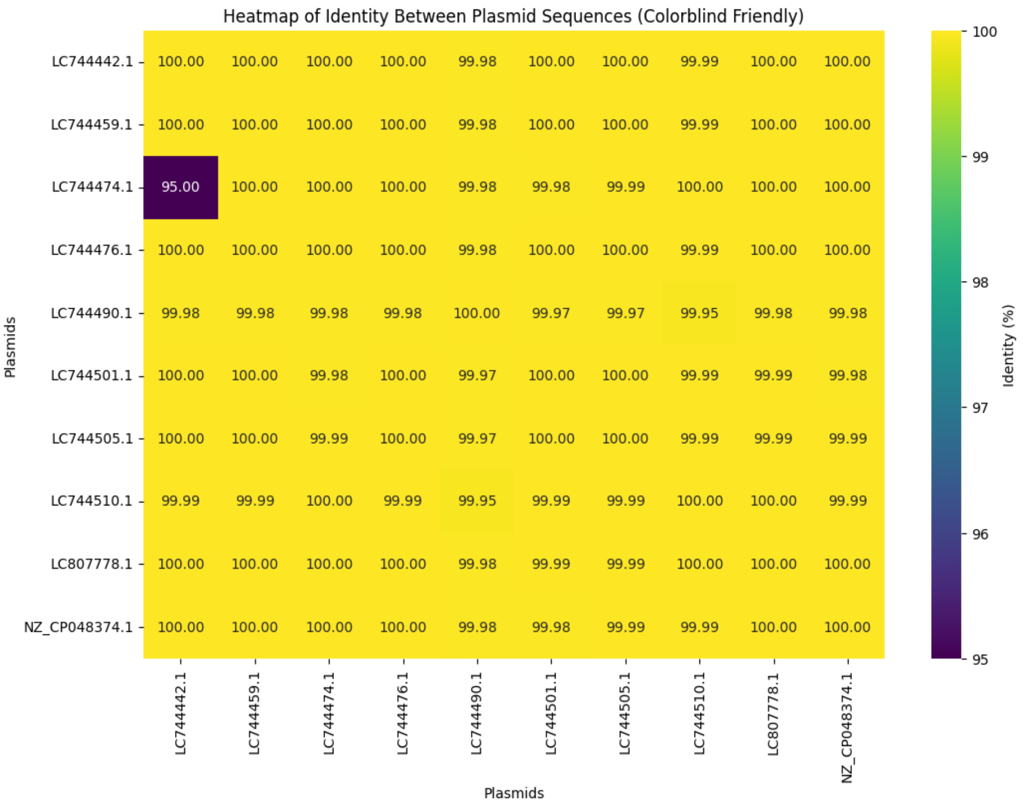
Heatmap of Identity Between Plasmid Sequences (Colorblind Friendly)
LC744442.1 100.00 100.00 100.00 100.00 99.98 100.00 100.00 99.99 100.00 100.00
LC744459.1 100.00 100.00 100.00 100.00 99.98 100.00 100.00 99.99 100.00 100.00
LC744474.1 95.00 100.00 100.00 100.00 99.98 99.98 99.99 100.00 100.00 100.00
LC744476.1 100.00 100.00 100.00 100.00 99.98 100.00 100.00 99.99 100.00 100.00
LC744490.1 99.98 99.98 99.98 99.98 100.00 99.97 99.97 99.95 99.98 99.98
LC744501.1 100.00 100.00 99.98 100.00 99.97 100.00 100.00 99.99 99.99 99.98
LC744505.1 100.00 100.00 99.99 100.00 99.97 100.00 100.00 99.99 99.99 99.99
LC744510.1 99.99 99.99 100.00 99.99 99.95 99.99 99.99 100.00 100.00 99.99
LC807778.1 100.00 100.00 100.00 100.00 99.98 99.99 99.99 100.00 100.00 100.00
NZ_CP048374.1 100.00 100.00 100.00 100.00 99.98 99.98 99.99 99.99 100.00 100.00
Plasmids
LC744442.1 LC744459.1 LC744474.1 LC744476.1 LC744490.1 LC744501.1 LC744505.1 LC744510.1 LC807778.1 NZ_CP048374.1
Plasmids
Identity (%)
100
99
98
97
96
95

Figure S7. **A**. Comparison among pECO-265-NDM-5 (used as a template), pM144-NDM-5, and the Pm21 assembly. BLAST analysis revealed no more than 20% coverage between Pm21 and pECO-265-NDM-5. Graphics were generated using Proksee, with relevant features annotated using CARD and mobileOG-DB. **B.** Comparison among pM40-NDM-5 (used as a template), pM366-NDM-5, pECO-265-NDM-5 and the Pm21 assembly. BLAST analysis revealed less than 15% with pM40-NDM-5 and p366-NDM-5. Only CDSs identified by gene names using Bakta were included in the graphic to simplify comprehension.

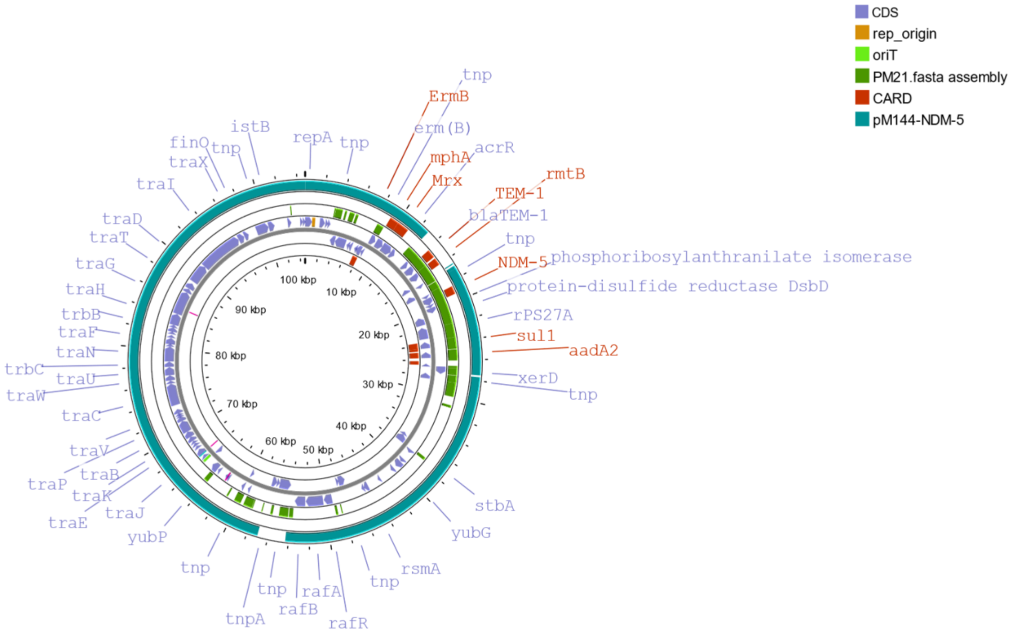

Fig S7 A *Escherichia coli* strain Ec265 plasmidpEco265-NDM-5, complete

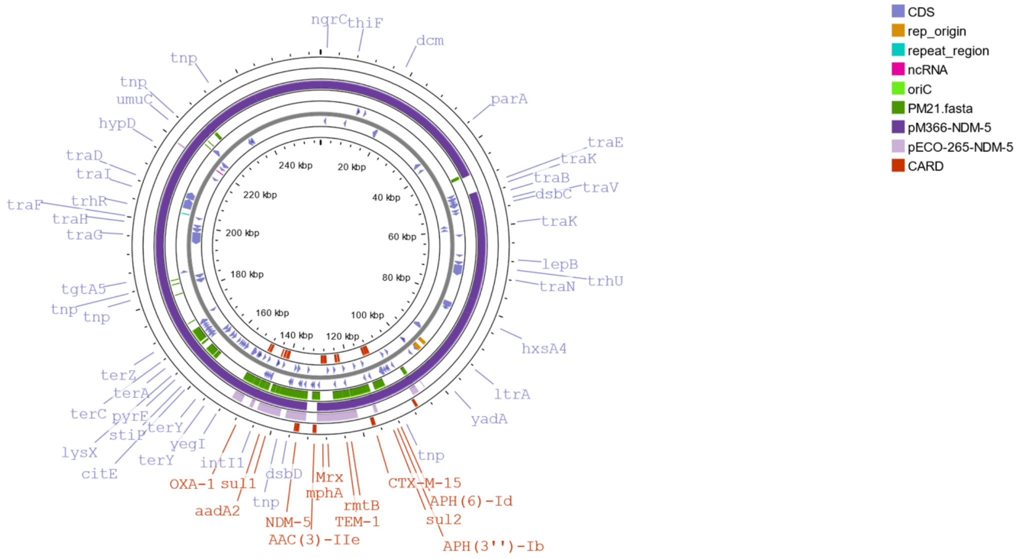

Fig S7B *Klebsiella pneumoniae* plasmid p40-NDM-5, complete

Figure S8. Nucleotide similarity and coverage of plasmid NZ_CP048374.1 (10,494 bp) with plasmids circulating in Argentina, including pM144-NDM-5, pEco265-NDM-5, pM40-NDM-5, and pM366-NDM-5, as well as with the Pm21 contig assembled using PlasmidSPAdes, all showing 100% coverage and nucleotide similarity.

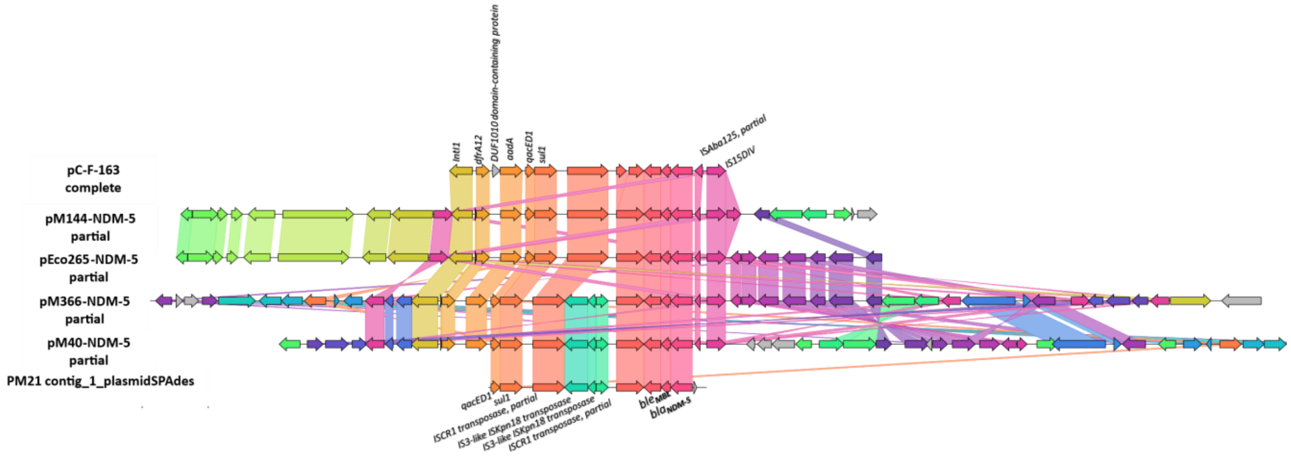

Supplement: Supplementary file 1 [file antibiotics-14-00557-s001.zip › antibiotics-3611648-supplementary.pdf]
